# Distinct Gut Microbiota Composition and Functional Category in Children With Cerebral Palsy and Epilepsy

**DOI:** 10.3389/fped.2019.00394

**Published:** 2019-10-01

**Authors:** Congfu Huang, Yinhu Li, Xin Feng, Dongfang Li, Xiuyun Li, Qiuxing Ouyang, Wenkui Dai, Genfeng Wu, Qian Zhou, Peiqin Wang, Ke Zhou, Ximing Xu, Shuaicheng Li, Yuanping Peng

**Affiliations:** ^1^Department of Pediatrics, Longgang District Maternity and Child Healthcare Hospital of Shenzhen City, Shenzhen, China; ^2^Department of Computer Science, City University of Hong Kong, Kowloon Tong, Hong Kong; ^3^Department of Microbial Research, WeHealthGene Institute, Shenzhen, China; ^4^Wuhan National Laboratory for Optoelectronics, Huazhong University of Science and Technology, Wuhan, China; ^5^School of Statistics and Data Science, NanKai University, Tianjin, China; ^6^Department of Healthcare, Longgang District Social Welfare Center, Shenzhen, China

**Keywords:** cerebral palsy, epilepsy, gut microbiota, co-occurrence network, KEGG functional category

## Abstract

Cerebral palsy (CP) and epilepsy are two interactive neurological diseases, and their clinical treatment can cause severe side-effects in children's development, especially when it involves long-term administration of antiepileptic drugs. Accumulating studies on the gut-brain axis indicated that the gut microbiota (GM), which participates in various neurological diseases, would provide a harmless therapeutic target for the treatment of CP and epilepsy. To explore the GM characteristics in children with both CP and epilepsy (CPE), we collected fecal samples from 25 CPE patients (CPE group) and 21 healthy children (Healthy group) for 16S rDNA sequencing. In this study, we discovered significantly higher microbial diversity in the CPE group compared to healthy group (*P* < 0.001). After selecting the top 15 most abundant genera in each group, we found significantly enriched *Bifidobacterium, Streptococcus, Akkermansia, Enterococcus, Prevotella, Veillonella, Rothia*, and *Clostridium IV* in the CPE group, and noticeably reduced *Bacteroides, Faecalibacterium, Blautia, Ruminococcus, Roseburia, Anaerostipes*, and *Parasutterella*. A GM co-occurrence network was also constructed, and negative correlations were discovered between *Bacteroides* and *Lactobacillus* (*r* = −0.768, *P* < 0.001, FDR < 0.001), as well as *Intestinibacter* and *Bifidobacterium* (*r* = −0.726, *P* < 0.001, FDR < 0.001). After KEGG annotation and functional enrichment, 24 functional categories exhibited different enrichment levels between the CPE and Healthy groups. The functions, associated with xenobiotics metabolism, immune system diseases, and neurodegenerative diseases, were enriched in the CPE group. Conversely, the functional categories related to the biosynthesis of secondary metabolites were reduced. Furthermore, the neurodegenerative diseases were mainly attributed to *Streptococcus*, while an increased risk of immune system diseases was associated with enriched *Akkermansia* in the CPE patients. Generally, this study characterized the GM in CPE patients, illustrated the microbial co-occurrence relationships, and detected the functional distributions of the bacteria.

## Introduction

Cerebral palsy (CP) refers to the non-progressive brain injuries that occur in the fetus or infant, and is a life-long physical disease with a childhood-onset (1, 2). Although it is defined by central dyskinesia and abnormal motor function (1, 2), CP is often accompanied with other complications, including epilepsy, orthopedic disorder, speech impairment, and neurobehavioral disorders (3). As one of the common complications, epilepsy occurs in 25–45% of CP patients (4), which is higher than the prevalence of 0.1–0.3% in all neonates (5). Epilepsy aggravates the brain injury in patients with CP (3, 4, 6). The current clinical therapy for CP patients with epilepsy is long-term physical rehabilitation training (2) and antiepileptic drug administration (or ketogenic diet) (7, 8). However, severe developmental side-effects have been reported in children following these clinical treatments (9). The rapid progress in gut microbiota (GM) research raise the possibility of detecting harmless therapies for these neurologic diseases through GM intervention (10, 11).

Increasing studies have suggested a close relationship between GM and various neurologic diseases, such as Autism Spectrum Disorder (ASD) (12), Parkinson's Disease (PD) (13), and Alzheimer's Disease (AD) (14). By secreting secondary metabolites into blood circulation, GM could stimulate the central nervous system (CNS), and affect the stress, cognition, and mood of hosts (10, 11). For example, De Angelis et al. reported increased *Clostridia* species in ASD patients (12), which might repress the activation of enterochromaffin cells and the secretion of serotonin (15). Additionally, decreased *Prevotellaceae* and increased *Lactobacilliaceae*, which relate to neuroinflammation, were discovered in neurodegenerative diseases, such as PD (13). In Gan Xie et al.'s study, increased Proteobacteria and Cronbacteria were found in patients with epilepsy, and the role of GM in seizure controlling was hinted (16). Although GM has been investigated in various single neurologic diseases, its characteristics in patients with both CP and epilepsy (CPE) remain unexplored.

We recruited 25 CPE patients and 21 healthy children aged 3–18 years old to investigate the GM features in CPE. In addition to exploring the GM differences between the CPE and Healthy groups, we also aimed to: (I) evaluate the microbial correlations in children; (II) elucidate the alteration of GM functions in CPE patients and their corresponding intestinal commensals. We hope this study will enhance our understanding of the roles of GM in the pathogenesis of CPE, and provide a solid theoretical basis for microbial intervention in CPE patients.

## Materials and Methods

### Ethics Statement

This study was approved by the Ethics Committee of The Hospital of Maternal and Child Health (Longgang, China) under registration number LGFY2017005. As the guardian of the CPE patients, the Longgang District Social Welfare Center provided written informed consent and volunteered their children for investigation for scientific research, as well as the parents of the healthy children.

### Participant Recruitment

The CPE patients were recruited from the Longgang District Social Welfare Center, and diagnosed by the Department of Neurology, The Hospital of Maternal and Child Health (Longgang, China) with the following inclusion criteria: (I) older than 3 years of age and younger than 18 years of age; (II) the patients were diagnosed with clear clinical manifestations of both CP and epilepsy in accordance with the diagnosis guidelines (17, 18). Healthy children were selected from those who passed physical examinations by the Department of Physical Examination, The Hospital of Maternal and Child Health (Longgang, China) with the following standards: (I) older than 3 years of age and younger than 18 years; (II) no allergic history (e.g., food allergy, AD, and asthma); (III) no hereditary diseases (e.g., thalassemia, hereditary deafness, and phenylketonuria); (IV) no metabolic diseases (e.g., obesity, diabetes, and rheumatoid arthritis). Moreover, the subjects were excluded from the study if they have been exposed to antibiotics, probiotics, or proton pump inhibitors 2 weeks before fecal sample collection. Finally, 25 CPE patients and 21 healthy children were enrolled between Aug. 2017 and Dec. 2017 (Supplementary File 1).

### Fecal Sample Collection

Fresh stool samples were collected from CPE and healthy subjects during the clinical examination using sample swabs [iClean, Huachenyang (Shenzhen) Technology Co., LTD, China], and these were stored in sterilized tubes (62-558-201, SARSTEDT AG & Co. KG, Germany). Then the fecal samples were transferred to a −80°C freezer for long-term storage within 30 min.

### DNA Extraction, Library Construction, and Sequencing

In compliance with the protocols of E.Z.N.A.® Soil DNA Kit (Omega Bio-tek, Norcross GA, U.S.A.), bacterial DNA was isolated from fecal samples. Using a PCR kit (AP221-02, TransGen Biotech, China), the hyper-variable V3-V4 regions of 16S rRNA were amplified with 338F and 806R primers. Then, the PCR products were prepared for sequencing library construction (TruSeq DNA PCR-Free kit, Illumina, San Diego CA, U.S.A.), and sequenced by MiSeq platform (Illumina, San Diego CA, U.S.A.) as 300 (nt) paired-end reads. Raw reads were uploaded to the NCBI Sequence Read Archive (SRA) Database (BioProject ID: PRJNA530084). After sequencing, the remaining DNA and fecal samples were stored in a −80°C refrigerator, and will be eliminated after 2 years, according to the regulations of standard biosecurity and institutional safety in The Hospital of Maternal and Child Health (Longgang, China).

### Taxonomical Annotation

The raw data was filtered as previously described (19). Based on at least 50 bases overlapping, the filtered reads were connected into tags, and the tags were clustered into operational taxonomic units (OTUs) with 97% similarity by USEARCH (v7.0.1090) (20). The taxonomical positions of OTUs were obtained by aligning their sequences to the RDP 16S rRNA database (trainset 16/release 11.5) (21) (Supplementary File 2).

### PERMANOVA to Evaluate the Influence of Physical Indices

PERMANOVA was carried out on GM composition of all samples to assess the impacts of physical indices, which are listed in Table 1 (22). The vegan package in R was applied with 9,999 permutations and Euclidean distances.

**Table 1:** Background information of the 46 study subjects.

	**Healthy (*n* = 21)**	**CPE patients (*n* = 25)**	***P*-value (PERMANOVA)**	**FDR**
Gender (male)	12	13	0.460	0.575
Age (month)	70.43 ± 20.93	108.13 ± 42.83	0.035	0.088
BMI	17.37 ± 4.60	12.35 ± 2.29	0.843	0.843
Anti-seizure drug usage	0	15	0.378	0.575

### Functional Prediction and Enrichment

With 16S rRNA OTUs profiling, the functional distributions of GM were acquired by PICRUSt with the default settings (23). For each sample, the abundance of KEGG Orthology (KO) was calculated, and the enriched functional categories on level III and level II of the KEGG database were detected (Supplementary File 3).

### Statistics

All statistical analysis was performed in R (version 3.4.1). The differentially enriched genera and functional categories between CPE patients and healthy children were detected by Wilcoxon rank-sum test (*P* < 0.05, using “wilcox.test” in R). The genera that were enriched in the CPE and Healthy groups were selected, and their associations were evaluated by Spearman correlations (using “cor” in R). The co-occurrence networks were visualized by Cytoscape software (v2.2.0) (24). Statistical results from the multiple tests were adjusted with Benjamini and Hochberg method (FDR < 0.05) using “p.adjust,” and were plotted using package “ggplot2” in R.

## Results

### Sample Characteristics and Data Output

A total of 25 CPE patients (CPE group) and 21 healthy children (Healthy group) were enrolled for fecal sample collection in this study (Table 1, Supplementary File 1). The high-quality reads from 16S rRNA sequencing were connected into 2,293,673 tags, and the number of OTUs was significantly higher in the CPE group than that in the Healthy group (*P* < 0.001): it ranged from 157 to 1,333 in the CPE group, and 109 to 242 in the Healthy group. After RDP database alignment, 341 genera of 21 phyla were identified in all samples (Supplementary File 2), and the number of genera was remarkably higher in the CPE group as compared to the Healthy group (*P* < 0.001): the averaged number was 152 ± 47 and 53 ± 8 in CPE and Healthy groups, respectively. Other than age, gender, body mass index (BMI), or anti-seizure drug exposure, health condition exhibited a significant impact on the GM differences between the CPE and Healthy groups (*P* < 0.001, FDR < 0.001, PERMANOVA analysis, Table 1).

### CPE Patients Held Discrepant GM Structure

**Figure 1:**
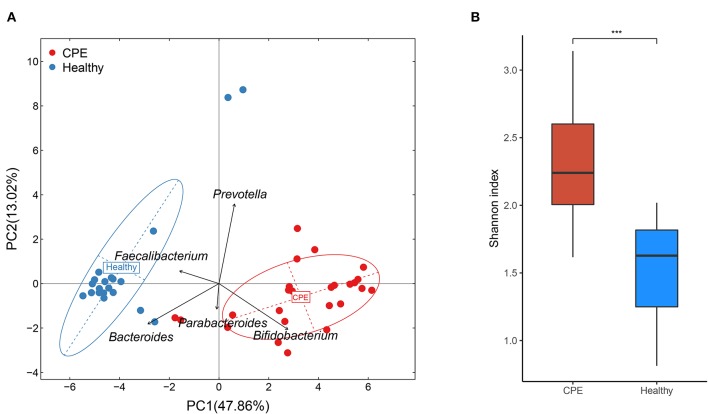
PCA and bacterial diversity analysis on fecal samples from CPE and healthy children. **(A)** PCA analysis indicated that samples from the Healthy group were clustered together, and they were apart from those of the CPE group. The GM of the participants was mainly dominated by *Bacteroides, Bifidobacterium, Prevotella, Faecalibacterium*, and *Parabacteroides*. **(B)** The microbial diversity was significantly higher in the CPE group (2.33 ± 0.43) than that in the Healthy group (1.49 ± 0.36). ^***^stands for *P*-value smaller than 0.001.

Principal component analysis (PCA) indicated that microbial samples from the CPE group were separated from those in the Healthy group, and the microbial samples were dominated by *Bacteroides, Bifidobacterium, Prevotella, Faecalibacterium*, and *Parabacteroides* (Figure 1A). Moreover, higher microbial diversity was identified in the CPE group (*P* < 0.001): the Shannon indexes were 2.33 ± 0.43 and 1.49 ± 0.36 in CPE and Healthy groups, respectively (Figure 1B).

**Figure 2:**
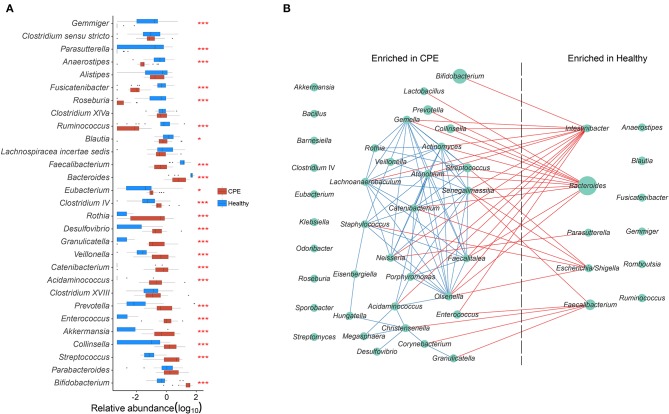
GM differences between CPE and Healthy groups, and bacterial co-occurrence network in all children. **(A)** Top 15 abundant genera were selected from CPE and Healthy groups, and their abundances were compared between these two groups. * and *** stand for the *P*-value smaller than 0.05 and 0.001. Healthy and CPE groups were represented with blue and red boxes, respectively. **(B)** The genera enriched in CPE and Healthy groups were selected, and their co-occurrence network was constructed. The blue and red edges suggest the positive and negative correlations, respectively, while the diameter of the circle is proportional to the relative abundance of the genera.

The top 15 most abundant genera from the CPE and Healthy groups were selected, and 23 of them were differentially enriched between these two groups (Figure 2A). Compared to the Healthy group, the CPE group contained higher proportions of *Bifidobacterium* (29.59 ± 15.07%, *P* < 0.001, FDR < 0.001), 0.001), *Streptococcus* (4.70 ± 3.61%, *P* < 0.001, FDR < 0.001), *Akkermansia P* < 0.001, FDR < 0.001), *Enterococcus* (1.88 ± 2.49%, *P* < 0.001, FDR < 0.001), FDR < 0.001), *Prevotella* (1.86 ± 2.52%, *P* < 0.001, FDR < 0.001), *Veillonella* (0.98 ± 1.21%, *P* < 0.001, FDR < 0.001), *Rothia* (0.62 ± 0.82%, *P* < 0.001, *P* < 0.001, FDR < 0.001), and *Clostridium IV* (0.60 ± 1.35%, *P* < 0.001, FDR < FDR < 0.001) (Figure 2A). In contrast, the relative abundances of *Bacteroides* (10.94 ± *P* < 0.001, FDR < 0.001), *Faecalibacterium* (0.78 ± 0.82%, *P* < 0.001, FDR < 0.001), *Blautia* (1.44 ± 2.68%, *P* = 0.022, FDR = 0.037), *Ruminococcus* (0.01 ± 0.02%, *P* < 0.001, FDR < 0.001), *Roseburia* (0.00 ± 0.00%, *P* < 0.001, FDR < 0.001), *Anaerostipes* (0.04 ± 0.06%, *P* < 0.001, FDR < 0.001), and *Parasutterella* (0.00 ± 0.00%, *P* < 0.001, FDR < 0.001) were notably decreased in CPE patients (Figure 2A).

A GM co-occurrence network was constructed based on the differentially enriched bacteria between the CPE and Healthy groups (Figure 2B). *Bacteroides* was negatively correlated with *Lactobacillus* (*r* = −0.768, *P* < 0.001, FDR < 0.001) and *Prevotella* (*r* = −0.722, *P* < 0.001, FDR < 0.001), while *Intestinibacter* was negatively correlated with *Bifidobacterium* (*r* = −0.726, *P* < 0.001, FDR <0.001) and *Enterococcus* (*r* = −0.707, *P* < 0.001, FDR = 0.002) (Figure 2B). On the other hand, complex positive correlations were unveiled among bacteria that were enriched in CPE patients. Positive relationships were discovered between *Streptococcus* and *Actinomyces* (*r* = 0.833, *P* < 0.001, FDR < 0.001), *Actinomyces* and *Veillonella* (*r* = 0.811, *P* < 0.001, FDR < 0.001), *Veillonella* and *Staphylococcus* (*r* = 0.682, *P* < 0.001, FDR = 0.009), and *Staphylococcus* and *Catenibacterium* (*r* = 0.790, *P* < 0.001, FDR < 0.001).

### Altered GM Function in the CPE Patients Was Correlated With Their Dysbiotic GM

**Figure 3:**
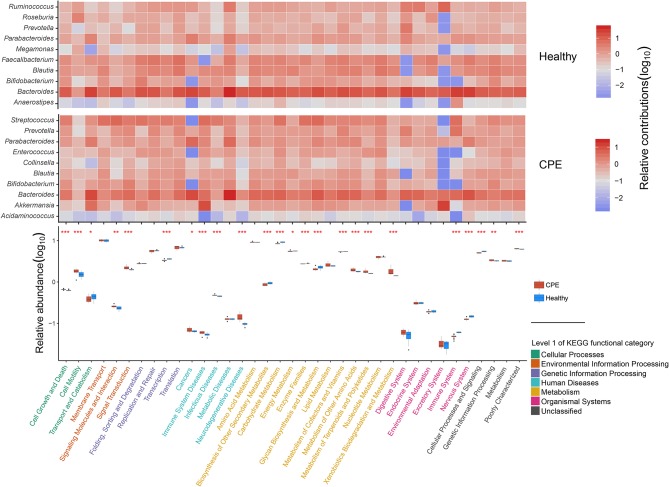
Distribution of functional categories for GM in CPE and Healthy groups. In the heatmap, the contributions of the top 10 genera on 37 KEGG level II functional categories were detected in CPE and Healthy groups, respectively. The deeper red square means the genera contribute to the functional category importantly, while the deeper blue square means the functional category obtained less contribution from the genera. In the box plot, the enriched pathways were compared between CPE and Healthy groups, and their *P*-values were indicated by asterisks. *, **, and *** stand for the *P*-value smaller than 0.05, 0.01, and 0.001, respectively. Functional classifications of KEGG level I were suggested by different colors, while the blue and red boxes represented Healthy and CPE groups, respectively.

Using PICRUSt software and the KEGG database, we obtained 6,909 KEGG orthologous groups (KOs) from all the samples (Supplementary File 3). All pathways collapsed into 37 functional categories at KEGG level II, and 24 of them were differentially enriched between the Healthy and CPE groups (Figure 3). The enriched functional categories in the CPE group included “Signal transduction” (2.22 ± 0.20%, *P* < 0.001, FDR < 0.001) and “Xenobiotics biodegradation and metabolism” (1.78 ± 0.30%, *P* < 0.001, FDR < 0.001) (Figure 3). By contrast, the proportions of microbial genes involved in “Biosynthesis of in “Biosynthesis of other secondary metabolites” (0.86 ± 0.06%, *P* < 0.001, system” (0.04 ± 0.00%, *P* < 0.001, FDR < 0.001), and “Nervous system” (0.12 ± 0.00%, *P* < 0.001, FDR < 0.001) were decreased in CPE patients, which corresponded with the increased risks increased risks of “Immune system diseases” (0.06 ± 0.00%, *P* < 0.001, FDR < 0.001) and “Neurodegenerative diseases” (0.14 ± 0.02%, *P* < 0.001, FDR < 0.001).

The increased risk of “Neurodegenerative diseases” in CPE patients was probably attributed to *Streptococcus, Parabacteroides*, and *Bacteroides* (Figure 3), among which *Streptococcus* was significantly enriched in the patients (*P* < 0.001, FDR < 0.001, Figure 2A). In healthy children, both *Faecalibacterium* and *Blautia* interacted with the “Nervous system” closely (Figure 3), but they decreased in CPE patients (Figure 2A). Moreover, the enriched *Akkermansia* and *Streptococcus* (Figure 2A) in the CPE group were the major contributors for the increased risk of “Immune system diseases” (Figure 3). Besides regulating the “Immune system,” *Bacteroides* participated in the metabolism of most nutrition in its host (Figure 3).

## Discussion

Though they are two different neurologic diseases, CP and epilepsy interact with each other closely: over 25% of CP patients are diagnosed with epilepsy (1), and epilepsy aggravates the motor and mental impairment of CP patients (3, 4, 6). An increasing numbers of studies on the gut-brain axis have suggested the potential impact of GM on various neurologic diseases (10, 11), though little is known regarding patients with CP and epilepsy. In this study, we characterized the GM features in CPE patients and their potential roles in the pathogenesis of CPE.

Compared with healthy children, the CPE patients exhibited lower proportions of *Anaerostipes, Faecalibacterium*, and *Bacteroides*. Jamie Joseph et al. have reported that *Anaerostipes* and *Faecalibacterium* spp. could produce butyrate with acetate (25), and butyrate could stimulate the differentiation of regulatory T (Treg) cells and relieve the neuroinflammation burdens (26). However, large amounts of acetate would accumulate in CPE patients with low abundances of *Anaerostipes* and *Faecalibacterium* spp., which could activate the parasympathetic nervous system (27) and trigger a seizure. In addition, the decreased *Bacteroides* would also reduce butyrate secretion and attenuate its neuroprotective effect in CPE patients (26). On the other hand, higher abundances of *Enterococcus, Bifidobacterium, Clostridium IV*, and *Akkermansia* were discovered in the CPE patients. *Enterococcus* could stimulate the production of serotonin in chromaffin cells and improve autistic behaviors through neuromodulation (28), whereas, excessive serotonin, most likely caused by the multiple drug treatments in the CPE patients, was harmful to the central nervous system (29). Although *Bifidobacterium* has been regarded as a beneficial microbe due to its ability to enforce the epithelial barrier, its higher abundance also indicated the immature microbiota in the patients (30). Furthermore, a higher abundance of *Clostridium* would aggravate microbial dysbiosis in CPE patients as a potentially pathogenic bacterium (31). With increasing non-dominated bacteria, dysbiotic GM in CPE patients exhibited higher microbial diversity than healthy children. Since complex competitive and synergistic relationships exist inside the GM (10), we further constructed the bacterial co-occurrence network to deepen our understanding of the GM features in CPE patients.

The co-occurrence network exhibited the negative correlations between bacteria enriched in healthy children (such as *Bacteroides, Escherichia*/*Shigella*, and *Intestinibacter*) and those enriched in CPE patients (such as *Bifidobacterium, Lactobacillus*, and *Enterococcus*). These antagonistic relationships indicated that the dominance of *Bacteroides* would repress the overgrowth of *Bifidobacterium* and *Lactobacillus* in individuals with mature GM (32). Probably due to its high diversity and abundance (33), *Bacteroides* participated in multiple metabolic pathways, including amino acid metabolism, energy metabolism, and lipid metabolism. Its abundance also reflected that *Bacteroides* could adapt to the diversified diet in adults, and provide energy and substrates to the host and other gut commensals.

Further analysis of microbial functions revealed the increased risks of immune systemic diseases and neurodegenerative diseases in CPE patients, and that neuroinflammation probably played a key role in the pathology of CPE (34). The elevated risk of immune system diseases was mainly attributed to the higher proportion of *Akkermnsia* in CPE patients. According to a previous report, the overgrowth of *Akkermnsia* would degrade mucin in mucous layers and increase the mucosal permeability (35), which enables more bacterial antigens to be exposed to the host immune system and trigger systematic immunoreactions in CPE patients. Moreover, the increased growth of *Streptococcus* would raise the levels of IL-6 and TNF-α (36), and induce neurodegenerative diseases by inducing neuroinflammation. Combined with the co-occurrence relationships among bacteria, we speculated that the mature GM was crucial for the development of the nervous system in children, and the clinical symptoms would possibly be relieved by GM interventions in CPE patients.

A limitation of the current research is the lack of a large number of patients with epilepsy. The comparison between CPE and epilepsy patients would better illustrate the roles of GM in the pathogenesis of CP and epilepsy, which would improve our understanding of the gut-brain axis. Additional work, as listed here, is also imperative in further studies: (I) performing large-cohort studies to verify the current findings; (II) exploring the alteration of intestinal metabolites in CPE patients, and their associations with microbiota; (III) recording the clinical improvement after treatment in CPE patients, and detecting their GM alterations.

In summary, this study presented the GM characteristics in CPE patients, illustrated the bacterial relationships inside the GM, and detected the functional distributions of GM in CPE patients. These findings suggest the roles of GM in the pathogenesis of CPE, and provide references for the bacterial adjuvant interventions in the treatment of CPE patients.

## Data Availability Statement

The datasets generated for this study can be found in NCBI Sequence Read Archive (SRA) Database, BioProject ID: PRJNA530084.

## Ethics Statement

This study was approved by the Ethics Committee of The Hospital of Maternal and Child Health (Longgang, China) under registration number LGFY2017005. As the guardian of the CPE patients, the Longgang District Social Welfare Center provided written informed consent and volunteered to receive investigation on their children for scientific research, as well as the parents of the healthy children.

## Author Contributions

YP, SL, KZ, and XX managed the project. CH, XL, QO, GW, and PW performed the fecal sampling and information collection. CH and QO prepared the DNA. YL, DL, and XF performed the bioinformatics analysis in this work. CH, YL, and DL interpreted the analysis results and wrote the paper. XF, WD, and QZ optimized the graphs. KZ, XX, SL, and YP guided statistical analysis and polished the article. All authors reviewed this manuscript.

### Conflict of Interest

The authors declare that the research was conducted in the absence of any commercial or financial relationships that could be construed as a potential conflict of interest.

## Supplementary Material

Supplementary File 1 Background information for enrolled subjects.

Supplementary File 2 Distribution of GM in all subjects at the phylum and genus levels.

Supplementary File 3 Distribution of bacterial functions in all subjects with KEGG database.
